# Soft tissue perineurioma of the tongue: report of a case and review of the literature

**DOI:** 10.1186/1477-7819-12-11

**Published:** 2014-01-13

**Authors:** Wen-lin Xiao, Ling-fa Xue, Yao-xiang Xu

**Affiliations:** 1 Department of Oral and Maxillofacial Surgery, The Medical School Hospital of Qingdao University, No. 1677, Wutaishan Road, Qingdao 266555, People’s Republic of China; 2 Department of Pathology, The Medical School Hospital of Qingdao University, No. 1677, Wutaishan Road, Qingdao, People’s Republic of China

**Keywords:** Perineurioma, Soft tissue, Tongue

## Abstract

Perineurioma is a rare benign tumor of the peripheral nervous system distinct from schwannomas and neurofibromas. It may be intraneural or extraneural (in the soft tissue). Extraneural soft tissue perineuriomas are uncommon; rare cases have been reported in the oral cavity. We present a case of soft tissue perineurioma in the tip of the tongue. The tumor was characterized by slender spindle cells, arranged in short fascicles or whorls, and focal areas showing a distinct storiform pattern. Tumor cells showed the immunohistochemical profile of perineurial cells, including epithelial membrane antigen. Smooth muscle actin, S-100, and CD34 were not expressed by the tumor cells. The tumor was surgically excised and in 2 years there has been no recurrence. Knowledge of the tumor in the oral cavity is important to reach a correct diagnosis and to avoid unnecessary aggressive local excision.

## Background

Perineuriomas are uncommon benign nerve sheath tumors composed exclusively of well-differentiated perineurial cells [1]. They are a group of clinically and histologically heterogenous tumors that have been divided into two main types: intraneural and extraneural. The extraneural soft tissue perineurioma is generally small and clinically manifests as a painless nodule; it occurs mostly in superficial soft tissue, and infrequently affects deep soft tissue [2-4]. Recent studies have established the histological appearance and clinical behavior of the soft tissue perineurioma. It is usually a well-circumscribed, firm mass with or without spontaneous pain or tenderness. Up to now, only four cases of perineuriomas located in the tongue have been described [1,5-7], all in young people. Among them, two cases were soft tissue perineuriomas and two cases were intraneural perineuriomas. We report the first case of soft tissue perineurioma occurring in the tip of the tongue of a 46-year-old woman and describe a tongue tumor with features of soft tissue perineurioma.

## Case presentation

A 46-year-old woman presented with a slow-growing, painless intraoral swelling of 3 years duration, which was located in the tip of her tongue. The patient was previously healthy and denied genetic or familial diseases. Physical examination revealed a well-delineated, elastic, hard, non-tender mass without pain.

**Figure 1:**
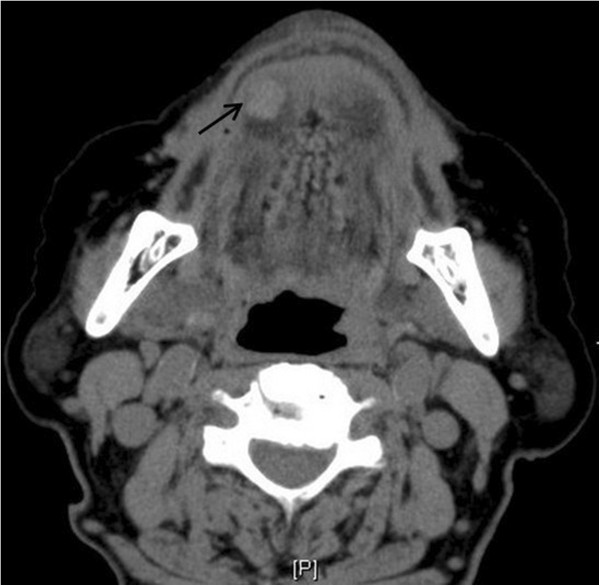
A spiral CT shows a round, well-circumscribed mass (arrow) in the right tip of tongue with homogeneous hypodensity compared with that of masseter.

A spiral computed tomography (CT) study showed a round very well-delineated soft tissue mass centered in the muscle of the tip of the tongue with homogeneous hypodensity compared with that of muscle (Figure 1).

**Figure 2:**
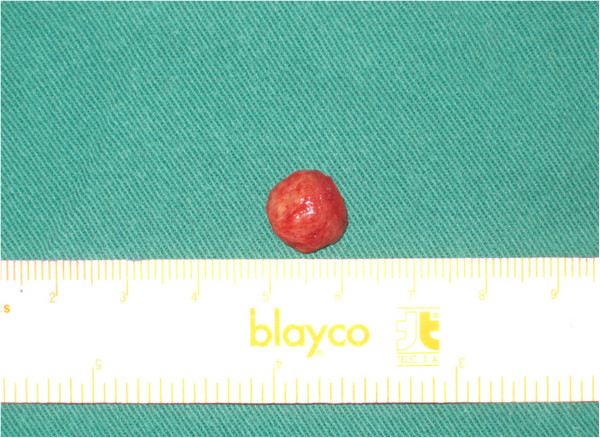
Gross view of specimen reveals a solid, whitish, homogeneous appearance, measuring 1.1 × 1.1 × 1 cm.

Surgical excision was performed. The tumor, along with about 1 mm of tongue tissue, was completely excised. The mass was excised, and the patient had no evidence of recurrence after 24 months follow-up. On gross examination, the surgically excised mass was circumscribed but not encapsulated. It measured 1.1 × 1.1 × 1 cm (Figure 2). The cut surface was grey-white and firm in consistency.

At low-power magnification, the tumor was well circumscribed but showed an unencapsulated proliferation of bland spindle cells. The spindle cells were characterized with tapered nuclei and delicate bipolar cytoplasmic processes. In some areas, these tumor cells had a distinct wavy appearance in the background of a diffusely collagenous and focally myxoid stroma.

**Figure 3:**
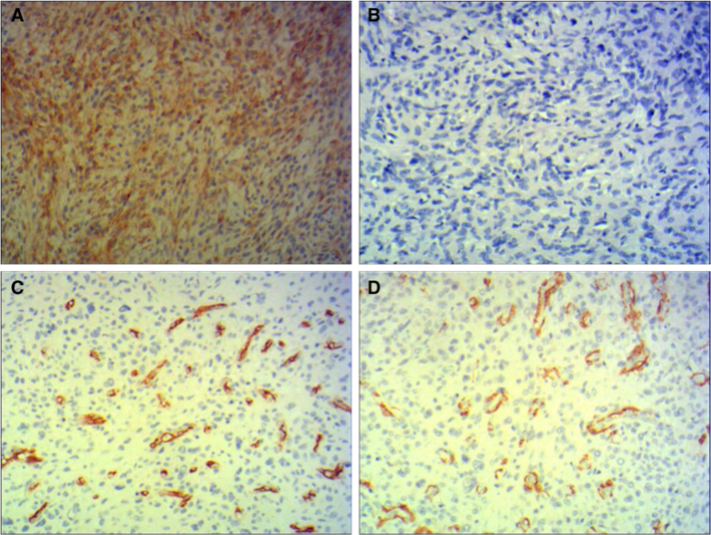
**Immunohistochemical results. (A)** Tumor cells stained positive for epithelial membrane antigen (original magnification × 200), and **(B)** complete negativity for S-100 (original magnification × 200). There was negative expression of **(C)** CD34 and **(D)** smooth muscle actin in all tumor cells (positive in reactive vessels) (original magnification × 200).

Immunohistochemically, these neoplastic cells stained positively for epithelial membrane antigen (EMA, Figure 3A), but the tumor cells failed to stain for S100 protein (Figure 3B), CD34 (Figure 3C) or smooth muscle actin (SMA, Figure 3D).

## Discussion

Perineuriomas are composed of perineurial cells, normally found as a thin layer around myelinated and unmyelinated peripheral nerves. Several tumors present perineurial cells to ensheath nerve fascicles; for example, nerve sheath myxoma, traumatic neuroma, and neurofibroma [4,8]. However, tumors formed purely of perineurial cells, perineuriomas, are uncommon and remain poorly recognized.

Extraneural soft tissue perineurioma is a benign peripheral nerve sheath neoplasm that often occurs in the subcutaneous tissues of the trunk and limbs as a painless solitary nodule or mass. Less frequently, it can be restricted to the dermis or occur in deep soft tissues. Some examples have also been described in the head and neck area [1]. Oral extraneural soft tissue perineuriomas are rare and tongue extraneural soft tissue perineuriomas are even rarer. The demographic and clinical features of our case and of four cases from the literature are summarized in Table 1. Despite initial observations suggesting that girls and women were more frequently affected, more recent studies have shown that soft tissue perineurioma has no sex predilection; it has been observed in both sexes [1,9]. Although the patient in our case is than 40 years old, several investigators have noted that extraneural soft tissue perineuriomas occur over a wide age range but are most prevalent in children and young adults [10].

**Table 1:** Clinical characteristic of reported tongue perineuriomas

**Reference**	**Variant**	**Age**	**Sex**	**Size (cm)**	**Follow-up (months)**
[1]	Extraneural	15	Female	1.2	N/A
[5]	Intraneural	26	Female	0.75	N/A
[6]	Extraneural	7	Female	2.0	ANED, 12
[7]	Intraneural	12	Male	0.6	ANED, 6
Present case	Extraneural	46	Female	1.1	ANED, 24

ANED, alive with no evidence of disease; N/A not available.

Light microscopic and immunohistochemical findings are required for a diagnosis of perineurioma. Histologically, soft tissue perineurioma shows variable histologic patterns and cellularity, consisting of spindled, wavy cells forming whorls, lamellar or storiform arrangements [1]. So far, academic circles have reached a consensus that in all perineuriomas, the neoplastic perineurial cells are EMA-positive and S-100 protein-negative [11]. In 1985, with the arrival of immunohistochemistry, Pinkus and Kurtin were first to note EMA expression in perineurial cells [12]. Nevertheless, EMA expression in soft tissue neoplasms is not restricted to perineuriomas; it can be detected in epithelioid sarcomas and in other mesenchymal neoplasms [13,14]. To diminish these problems, additional protein markers have recently been reported to be helpful in confirming the diagnosis of perineurioma, including S-100, CD34, SMA, and CD68 [1,4]. Cellular schwannoma cells stain positive for S-100, may stain positive or negative for CD34 and stain negative for EMA. Solitary fibrous tumor cells stain negative for S-100 and EMA, and stain positive for CD34. Benign fibrous histiocytoma or low-grade malignant fibrous histiocytoma cells stain negative for S-100, CD34, and EMA, and stain positive for CD68. Overall, immunohistochemical studies will help distinguish these three entities [1,4]. Smooth muscle actin has been reported to be positive in 50% of perineurioma cases; this indicates that it is also an important diagnostic marker. Based on previous research, immunohistochemical staining for perineurioma cells was performed using EMA, S-100, CD34, and SMA.

The etiology of perineuriomas is as yet unknown. The question has arisen as to whether it is an uncommon reactive lesion with proliferation of perineurial cells secondary to trauma or whether it is a true benign neoplasm. Because most cases, including the present case, could not show an association with a previous trauma [5], the extent of the perineurial proliferation has been shown to be clonal [15]. In particular, alterations on chromosome 22 have been reported in some of these patients; therefore, perineurioma is probably a neoplasm [16,17].

Because only 5% of the reported cases have shown recurrence [4], it seems that a complete local excision is the treatment of choice [5,18]. Finally, it is important to emphasize that it is essential for the final diagnosis of perineurioma to confirm the immunophenotype paralleling the normal perineurial cell (S-100 protein-negative and EMA-positive) [7].

## Conclusions

In summary, we have described a rare case of tongue perineurioma diagnosed by typical histologic features and unequivocal immunohistochemical expression of perineurial cell markers. Awareness of this unusual anatomical site for soft tissue perineurioma should enhance its proper recognition and allow delineation of the clinicopathologic profiles of tongue perineurioma.

## Consent

Written informed consent was obtained from the patient for publication of this case report and any accompanying images. A copy of the written consent is available for review by the editor-in-chief of this journal.

## Abbreviations

CT: Computed tomography; EMA: Epithelial membrane antigen; SMA: Smooth muscle actin.

## Competing interests

The authors declare that they have no competing interests.

## Authors’ contributions

WX obtained medical history, searched and reviewed the literature, drafted the manuscript, and edited the final version. LX obtained patient follow-up information, carried out the histopathological studies, and edited the final version. YX obtained medical history, provided diagnostic consultation, managed the patient, and edited the final version. All authors read and approved the final manuscript.
